# Multi-scale magnetic mapping of serpentinite carbonation

**DOI:** 10.1038/s41467-017-01610-4

**Published:** 2017-11-30

**Authors:** Masako Tominaga, Andreas Beinlich, Eduardo A. Lima, Maurice A. Tivey, Brian A. Hampton, Benjamin Weiss, Yumiko Harigane

**Affiliations:** 1 Department of Geology and Geophysics, Texas A&M University, College Station, TX 77845–3115 USA; 2 The Institute for Geoscience Research (TIGeR), Curtin University, Perth, 6845 Australia; 3 Department of Earth, Ocean and Atmospheric Sciences, The University of British Columbia, 2207 Main Mall, Vancouver, BC Canada V6T 1Z4; 4 Physics of Geological Processes (PGP), University of Oslo, Oslo, 0316 Norway; 5 Department of Earth, Atmospheric, and Planetary Sciences, Massachusetts Institute of Technology, 77 Massachusetts Avenue, Cambridge, MA 02139 USA; 6 Department of Geology and Geophysics, Woods Hole Oceanographic Institution, 266 Woods Hole Rd., Woods Hole, MA 02543 USA; 7 Department of Geological Sciences, New Mexico State University, Las Cruces, NM 88003 USA; 8 The National Institute of Advanced Industrial Science and Technology, 1–1–1 Umezono, Tsukuba, Ibaraki 305–8568 Japan

## Abstract

Peridotite carbonation represents a critical step within the long-term carbon cycle by sequestering volatile CO_2_ in solid carbonate. This has been proposed as one potential pathway to mitigate the effects of greenhouse gas release. Most of our current understanding of reaction mechanisms is based on hand specimen and laboratory-scale analyses. Linking laboratory-scale observations to field scale processes remains challenging. Here we present the first geophysical characterization of serpentinite carbonation across scales ranging from km to sub-mm by combining aeromagnetic observations, outcrop- and thin section-scale magnetic mapping. At all scales, magnetic anomalies coherently change across reaction fronts separating assemblages indicative of incipient, intermittent, and final reaction progress. The abundance of magnetic minerals correlates with reaction progress, causing amplitude and wavelength variations in associated magnetic anomalies. This correlation represents a foundation for characterizing the extent and degree of in situ ultramafic rock carbonation in space and time.

## Abstract (web summary)

Peridotite carbonation plays an important role in the carbon cycle. Here, the authors present a geophysical characterization of serpentinite carbonation from km to mm scale and confirm that the abundance of magnetic minerals provides a strong correlation with the overall carbonation reaction process.

## Introduction

Peridotite serpentinization and carbonation play important roles in facilitating large-scale cycling of volatiles between the atmosphere, hydrosphere, and lithosphere^1, 2^. The uptake of atmospheric and hydrospheric carbon during ultramafic rock carbonation particularly represents a natural analog to geologic carbon sequestration and is considered as one potential pathway to offset anthropogenic CO_2_ emissions into the Earth’s atmosphere^3–7^. Natural carbonation of ophiolite—alpine-type ultramafic rocks forms alteration assemblages known as ophicarbonate, soapstone, and listvenite. These different carbonation products differ in the composition of secondary sheet silicate phases and the abundance of carbonate and thus their bulk rock CO_2_ content. Listvenite is predominantly composed of carbonate and quartz and represents a desirable product during in situ CO_2_ sequestration in ultramafic formations. While natural ultramafic rock carbonation may take place over long time scales, its efficiency has yet to be proven on human time scales. Carbonation reaction parameters are extensively investigated by laboratory-scale hydrothermal experiments^8–11^, thermodynamic modeling^3, 12^. and natural analog studies^13–19^. However, a scheme that can delineate the carbonation reaction progress in situ has not yet been fully explored and upscaling of reaction parameters from small scale, controlled laboratory experiments to large scale, complex natural processes remains challenging^20–22^. Along the reaction path of hydrothermal alteration of ultramafic rock, silicate mineral replacement reactions concomitantly release Fe for incorporation into secondary oxide, sulfide, and carbonate phases^23, 24^. Of particular interest is the production and consumption of magnetite during reaction of ultramafic rock with hydrothermal fluids due to its strong influence on bulk rock magnetic properties^25^. If coherently observable at multiple scales, we propose that changes in rock magnetic properties related to peridotite serpentinization and subsequent carbonation can be linked to distinct steps along the reaction path, and hence that the reaction progress can be monitored by field magnetometry.

In this study, we investigate magnetic anomaly changes related to natural serpentinite carbonation using regional, outcrop-, and thin section-scale magnetometry coupled with microtextural analysis of mineral replacement reactions. The results show that the magnetic character of distinct alteration product assemblages changes in response to the stability of magnetic carrier phases. Progressive serpentinite carbonation is characterized by a transient increase in the magnetic field strength during intermittent carbonation. The final alteration product is almost devoid of magnetic carrier phases and thus characterized by a very weak magnetic field strength. These findings indicate that magnetic field measurements can be used to detect carbonation fronts in the field and to monitor reaction progress in space and time.

## Results

### Field relationships

**Fig. 1:**
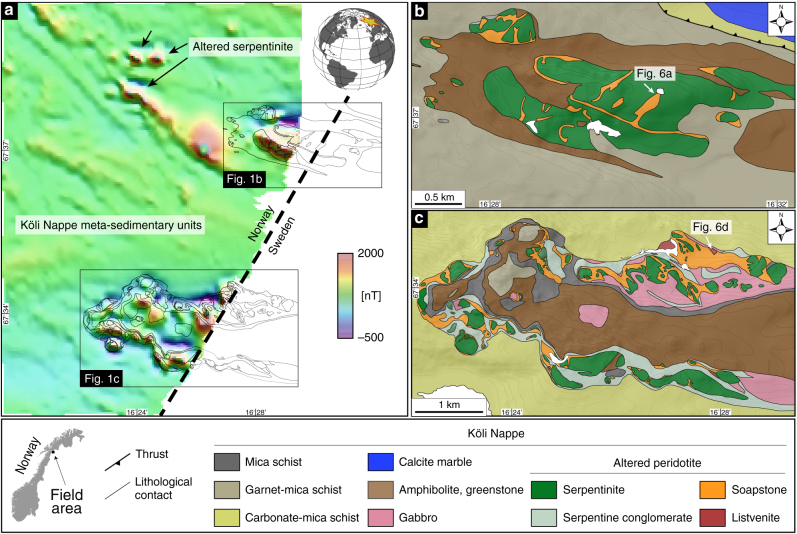
Aeromagnetic anomaly and geology of the Linnajavri area. **a** Magnetic anomaly map from the DRAGON aeromagnetic survey of the Linnajavri area showing the location of pristine and altered serpentinite bodies. **b**, **c** Geological maps of the Linnajavri Ultramafic Complex (LUC) northern **b** and southern parts **c** ^13, 27^. The location of outcrop-scale magnetic survey lines (Fig. 6) is indicated in **b**, **c**. Geological and geophysical data do not exist for the blank area

Widespread and near perfect exposure of naturally carbonated serpentinite at the Linnajavri Ultramafic Complex (LUC) in the Upper Allochthon (Köli nappe) of the Norwegian Caledonides represents an excellent natural laboratory to study the effects of ultramafic rock carbonation on changes in geophysical properties at the field scale (Fig. 1). The LUC represents a dismembered ophiolite complex separated from the Precambrian granitic basement by an up to ~6 km thick pile of greenschist facies metamorphosed sedimentary rocks. Weichselian glaciation of Scandinavia polished the rock surfaces, resulting in weathering rinds of < 2 mm thickness.

**Fig. 2:**
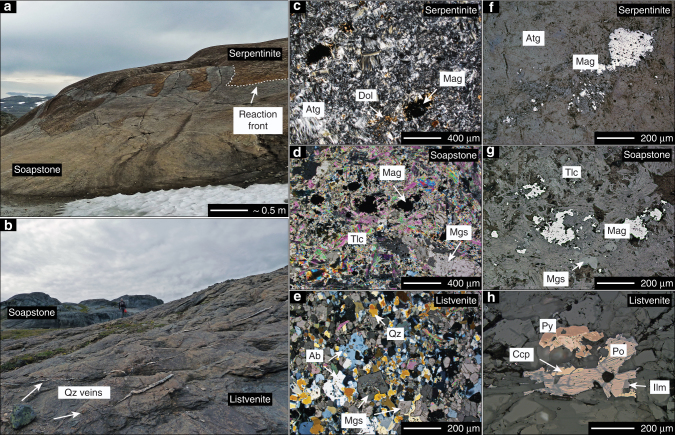
Field and microtextural relationships of carbonated serpentinite. **a** Field image of a sharp soapstone reaction front locally following fractures in the serpentinite. **b** Typical appearance of listvenite in the field with abundant quartz veinlets in front of massive soapstone (person for scale). **c**–**e** Representative micrographs of serpentinite, soapstone, and listvenite mineral assemblages in cross-polarized transmitted light. **f**–**h** Reflected light micrographs of oxide and sulfide phases in serpentinite, soapstone, and listvenite. Magnetite in serpentinite and soapstone is present as large grains and as fine grained matrix constituent with grain-sizes between ~10 µm and ~500 µm. Listvenite contains in most cases only relict amounts of magnetite and sometime additional pyrite and chalcopyrite together with minor pyrrhotite. Mineral abbreviations follow Whitney and Evans^49^

**Fig. 3:**
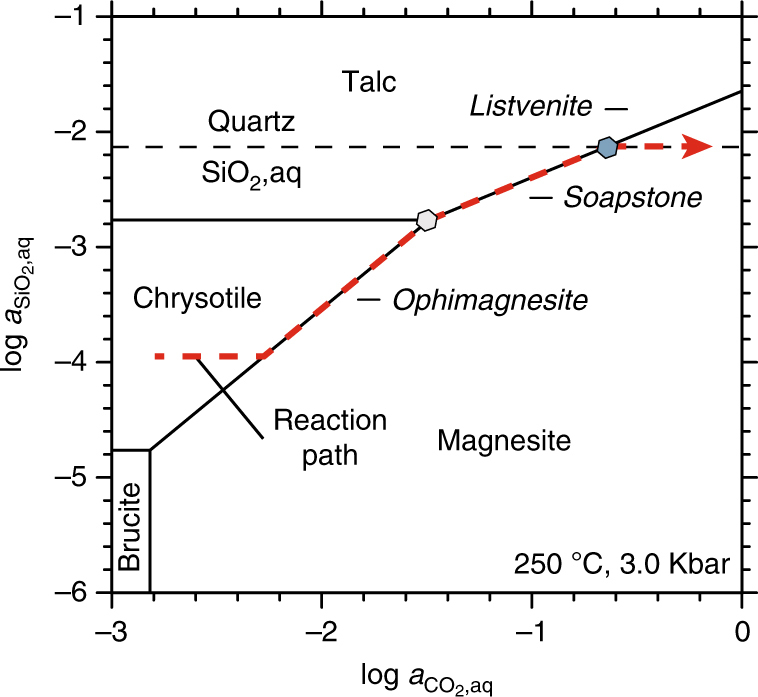
Reaction path of serpentinite carbonation. CO_2_–SiO_2_ activity diagram in the system MgO–SiO_2_–H_2_O–CO_2_ showing the reaction path of progressive serpentinite carbonation resulting in ophimagnesite (serpentine + magnesite), soapstone (talc + magnesite), and listvenite (quartz + magnesite) formation at constant pressure and temperature. Hexagon symbols mark CO_2_ activity values used in Fig. 9. Mineral stability fields are calculated using the computer program Supcrt and thermodynamic database dprons96.dat^50^, quartz saturation is based on the thermodynamic data of Rimstidt^51^. The estimated pressure of 3 kbar is based on a normal thermobaric gradient in a slightly thickened crust (12 bar/°C)^52^. The same diagram calculated for alteration temperatures of 180 °C and 300 °C is included in the supplement (Supplementary Fig. 1)

Previous studies have documented that infiltration of CO_2_-bearing fluid resulted in the formation of distinct zones of minor serpentine-magnesite (ophimagnesite; ~6.9 wt% CO_2_), extensive talc-magnesite (soapstone; ~14.9 wt% CO_2_) and quartz-carbonate (listvenite; ~29.3 wt% CO_2_) assemblages from completely serpentinized peridotite during the Caledonian orogeny (Fig. 2a, b; Supplementary Table 1)^13, 26, 27^. The soapstone alteration zone is separated by ubiquitously visible sharp reaction fronts, which are indicative of infiltration-driven metasomatic replacement^12, 13, 28, 29^. At outcrops, serpentinite carbonation is controlled by structural permeability and concentrated along the basal contact of the ophiolite with the underlying sediments and along faults within the serpentinite. Soapstone alteration zones reach several hundred meters into the ophiolite, while fracture-related alteration selvages in serpentinite are usually < 3 m wide (Fig. 2a). Formation of the ophimagnesite assemblage is typically restricted to a few centimeters in front of some soapstone fronts. Listvenite is exclusively present above the basal thrust and separated from uncarbonated serpentinite by soapstone (Figs. 1c, 2b). The zonal distribution of the different alteration assemblages indicates that the reaction fronts progressively moved from the basal thrust into the ophiolite, thereby replacing the earlier formed assemblage. Previous work indicated isothermal soapstone and listvenite formation near 250–300 °C in response to different fluid CO_2_ activities at a given pressure and temperature^13^ (Fig. 3). Thus, the distribution of alteration zones indicates a decreasing fluid CO_2_ activity from the inferred fluid inlet at the basal thrust into the ophiolite resulting from continuous dilution of the CO_2_-bearing alteration fluid due to serpentine breakdown and carbonate precipitation along the flow path.

### Sample petrography and carbonation reactions

The field relationships are consistent with microtextural analysis of mineral replacement reactions. Serpentinite represents the least altered rock type at the LUC and consists of more than 95 vol.% of antigorite together with isolated talc–dolomite intergrowths that are pseudomorphically replacing primary clinopyroxene, together with minor tremolite, Cr-spinel, and magnetite (Fig. 2c, f). Soapstone fronts are sharp on the outcrop and thin section scales, and are defined by the complete breakdown of antigorite to form talc and magnesite (Fig. 2a, d, g). In a Fe-free model system, the soapstone forming reaction can be simplified to:

1 $${\displaystyle{2{\rm{Mg}}_{3}{\rm{Si}}_{2}{\rm{O}}_{5}\left({{\rm{OH}}} \right)_{4}}\atop{{\mathrm{Serpentine}}}} {\displaystyle+ {3}{\mathrm{CO}}_{2,{\mathrm{aq}}} \to {{3{\mathrm{MgCO}}_{3}}}\atop {{\mathrm{Magnesite}}}} \\ \\ + {\displaystyle{{{\mathrm{Mg}}_{3}{\mathrm{Si}}_{4}{\mathrm{O}}_{10}\left( {{\mathrm{OH}}} \right)_{2}}}\atop{{\mathrm{Talc}}}} + {3}{\mathrm{H}}_{2}{\mathrm{O}}.$$

**Fig. 4:**
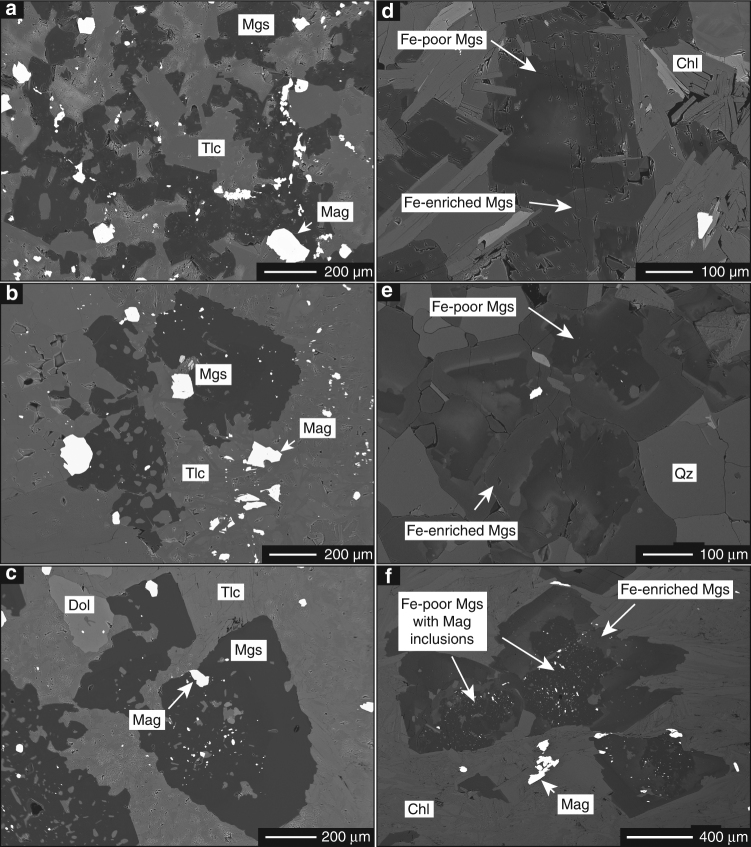
Magnesite growth textures. Back-scattered electron (BSE) images showing magnesite textures in soapstone **a**–**c** and listvenite **d**–**f**. Anhedral to subhedral magnesite in the soapstone frequently contains magnetite inclusions, whereas euhedral magnesite rims related to listvenite formation are in most cases devoid of magnetite. The zonation of listvenite-magnesite is caused by elevated Fe/Mg in the euhedral rim

The soapstone assemblage is stable at fluid CO_2_ activities between those stabilizing ophimagnesite (lower *a*CO_2_) and listvenite (higher *a*CO_2_) (Fig. 3)^12, 13, 28^. In the soapstone, anhedral to subhedral magnesite is enclosed in a matrix composed of mainly talc and minor clinochlore (Fig. 4a–c). Magnetite is present as a matrix component, inclusions in magnesite and rims on large (>100 µm) oxide grains that commonly comprise a Cr-spinel or Cr-magnetite core. Breakdown of Fe-bearing serpentine allows for magnetite formation in the soapstone in addition to magnetite inherited from the precursor serpentinite. Listvenite formation proceeds by dissolution of talc and precipitation of quartz and additional magnesite (in the simplified Fe-free system):

2 $${{{\mathrm{Mg}}_3{\mathrm{Si}}_4{\mathrm{O}}_{10}\left( {{\mathrm{OH}}} \right)_2}\atop{{\mathrm{Talc}}}} {+ 3{\mathrm{CO}}_{2,{\mathrm{aq}}} \to {{3{\mathrm{MgCO}}_3}\atop{\mathrm{Magnesite}}}} {+ {{{4{\mathrm{SiO}}_2}\atop{{\mathrm{Quartz}}}}}} + {\mathrm{H}}_2{\mathrm{O}}.$$

**Fig. 5:**
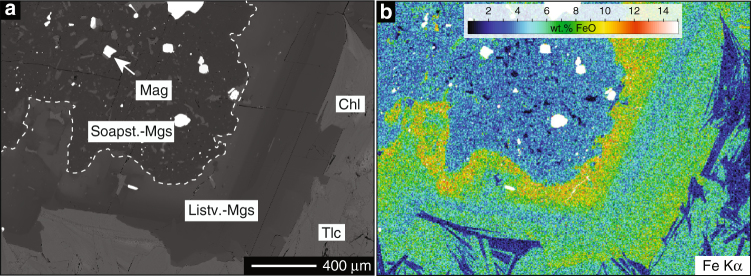
Compositional zoning in listvenite magnesite BSE image. **a** and quantitative element map of zoned magnesite **b** in the listvenite showing the increase in FeO in the euhedral magnesite rim overgrowing a low FeO, magnetite inclusion rich, anhedral magnesite core related to earlier soapstone formation

In contrast to soapstone and serpentinite, listvenite contains additional mica (biotite, Cr-muscovite), chlorite, albite, tourmaline, and sometimes sulfide phases (Fig. 2e, h). Magnetite is significantly less abundant or absent. Soapstone and listvenite magnesite are texturally and compositionally distinct. The reaction textures imply that euhedral listvenite magnesite overgrows preexisting, anhedral soapstone magnesite (Fig. 4). The core-rim interface resembles the crystal shape of magnesite in the soapstone. The core region of these composite grains frequently contains magnetite inclusions and has a high X_Mg_ (X_Mg_ = Mg/(Mg + Fe) ≈ 0.93), whereas the euhedral magnesite rim is devoid of magnetite inclusions and distinctly enriched in Fe (X_Mg_ ≈ 0.86) (Fig. 5)^13^.

### Geophysical field survey

We conducted a multi-scale geophysical investigation across the carbonation fronts by integrating aeromagnetic data (~10 km), total magnetic field and magnetic susceptibility surveys at outcrops (10−100 m), and magnetic mapping of thin sections (µm) from drill core samples acquired along the survey lines. The crustal-scale aeromagnetic field data of the LUC region were obtained by the Norwegian Geological Survey’s 1991 DRAGON aeromagnetic survey using a Scintrex MEP410 cesium magnetometer with an average survey altitude of 60 m and line spacing of 200 m.

**Fig. 6:**
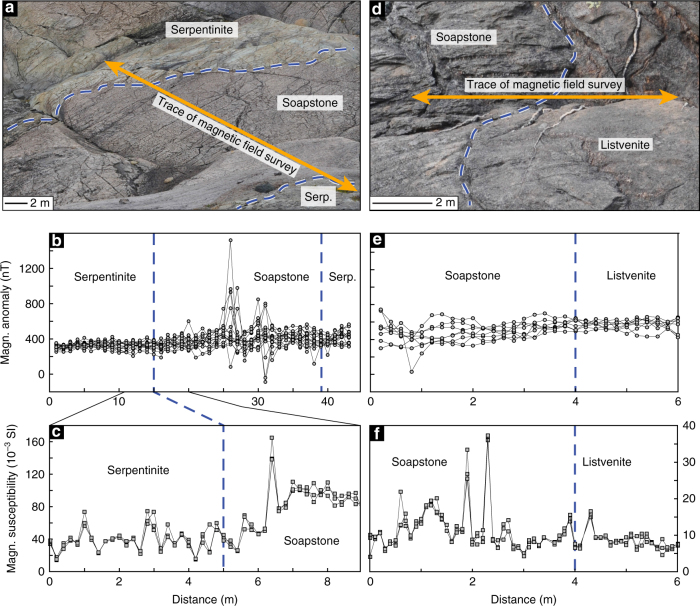
Outcrop magnetic anomaly measurements. Outcrop scale total field and magnetic susceptibility mapping across the serpentinite-soapstone interface **a** and the soapstone-listvenite interface **d**. Total magnetic field values across the serpentinite-soapstone interface were acquired along 16 transects (along the orange line in **a**) with a sampling interval and line spacing of 0.6 m and 0.2 m, respectively **b**. A total of three magnetic susceptibility measurement profiles were acquired along a portion of the same transect **c**. Total magnetic field values across the soapstone-listvenite interface were acquired along 8 transects (along the orange line in **d**) with a sampling interval and line spacing of 0.2 m **e**. A total of three magnetic susceptibility measurement profiles were acquired along the same transect **f**

The aeromagnetic total field of the Linnajavri region exhibits small provinces with distinctive high amplitude, short-wavelength (<100 m) anomalies in contrast to the surrounding metasedimentary units, which exhibit only weak field values and minimal amplitude variations. The locations of high amplitude, short-wavelength anomalies coincide with the distribution of mapped ultramafic complexes (Fig. 1a–c). Outcrop-scale total field magnetic anomaly surveys were conducted using a high-precision Applied Physics fluxgate magnetic sensor across serpentinite soapstone (Fig. 6a, b) and soapstone- listvenite (Fig. 6d, e) reaction fronts. All total field aeromagnetic and outcrop-scale total field magnetic anomaly surveys were corrected for the International Geomagnetic Reference Field model–12^30^. Magnetic susceptibility measurements were conducted along the magnetometry transects using a Terra–TK04 susceptometer (Fig. 6c, f). In contrast to total field magnetic anomaly measurements, susceptibility measurements only capture the surface (~2 cm) mineralogy of the surveyed formation.

Changes in total field magnetic anomaly amplitudes over measurement profiles originate from the combination of both natural and induced remanent magnetization (NRM) of the rock formation, whereas changes in magnetic susceptibility values represent the abundance and chemical composition of magnetic carriers. In the serpentinite and soapstone samples magnetite represents the only magnetic carrier phase, while magnetite is absent in listvenite or present in only minor amounts with occasional additional iron sulfide phases (pyrite, chalcopyrite, and pyrrhotite) (Fig. 2f–h). Changes in total field anomaly and susceptibility wavelengths represent magnetic boundary spacing and the presence of different alteration assemblages separated by reaction fronts. Overall, the magnetic field measurements show distinctly higher amplitudes and shorter wavelengths in the soapstone relative to the adjacent serpentinite and listvenite formations (Fig. 6).

Outcrop-scale total magnetic field anomaly profiling also documents magnetic anomaly contrasts between the ultramafic and sedimentary units with less ambiguity. The “background” magnetic anomalies over the thick metasedimentary units are almost zero after the regional field correction, whereas both magnetic anomaly and susceptibility profiles across the serpentinite-soapstone and soapstone-listvenite fronts show marked changes in anomaly amplitudes and wavelengths. Along an idealized reaction path from low to high *a*CO_2_, magnetic anomaly amplitudes are high in serpentinite, and even higher in soapstone, but minimal in listvenite with little variations in amplitude and wavelength (Fig. 6b, e). Magnetic susceptibility profiles across serpentinite soapstone and soapstone-listvenite fronts follow this same trend in amplitude and wavelength variation (Fig. 6c, f), consistent with previously reported magnetic susceptibility observations^10^.

### Thin section SQUID microscopy

Magnetic field mapping at sub millimeter-scale spatial resolution on representative thin sections from serpentinite, soapstone, and two listvenite samples acquired from two different localities at the LUC were conducted using a scanning superconducting quantum interference device (SQUID) microscope at the Massachusetts Institute of Technology Paleomagnetism Laboratory. The instrument’s magnetic field sensitivity is ~0.01 nT and measures the vertical component of the magnetic field in a rectangular grid of positions above thin sections^31^. NRM fields were measured on four thin sections representing each lithology at a sensor-to-sample distance of 170 µm and with 85 µm line spacing. At the same imaging resolution, we also performed anhysteretic remanent magnetization (ARM) field measurements to assess the distribution of magnetic carriers within the samples at fine spatial scale. After full alternating-field demagnetization of the samples, we imparted ARMs with a bias field of 100 μΤ and a peak alternating field of 260 mT to activate magnetic sources in the low to high coercivity range and assess the capacity of the sample to acquire magnetization.

**Fig. 7:**
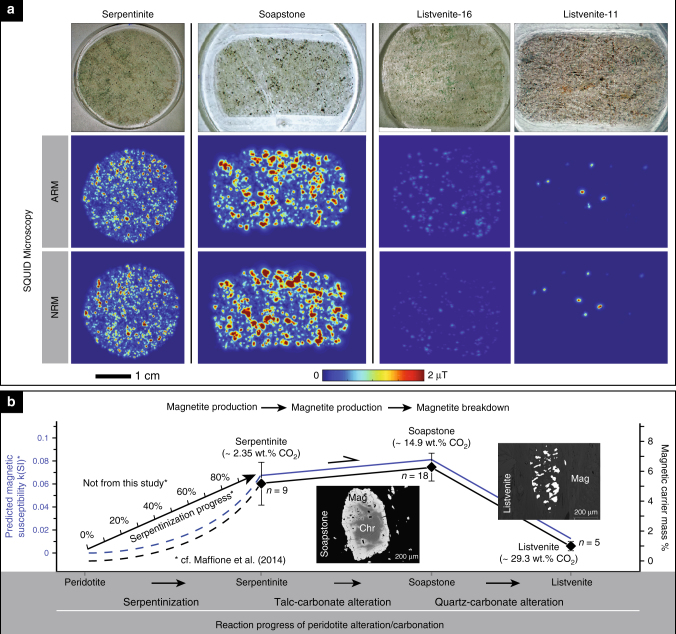
Thin section magnetic anomaly measurements. **a** NRM and ARM (with the peak alternating field of 260 mT and the DC bias field of 100 μΤ) in 30 µm-thickness thin sections of serpentinite, soapstone and two listvenite samples acquired by SQUID microscopy. The samples are mounted on 1-inch discs. Shown is the total magnetic field at a height of 170 µm above the samples. For comparison, the SQUID images are presented with the color scale of 0–2 µT (Note the grains in the serpentinite and soapstone samples are mostly >2 µT). Listvenite samples show significantly weaker magnetic signal strength compared to serpentinite and particularly soapstone. Relict magnetite grains in the listvenite are mostly present as inclusions in magnesite cores and hence passivated from reaction during listvenite formation. **b** Schematic of changes in magnetite abundance and predicted magnetic field strength changes versus reaction progress of ultramafic rock serpentinization^43^ and carbonation. Magnetic susceptibility values are based on Maffione et al.^43^ and may differ in other alteration settings depending on rock composition and alteration temperature. Diamond symbols show the calculated bulk rock content of magnetite in the different alteration zones assuming bulk rock Fe^3+^ is exclusively present in magnetite (Supplementary Table 1). Error bars denote the 1σ standard deviation of averaged bulk Fe^3+^ weight fractions

**Fig. 8:**
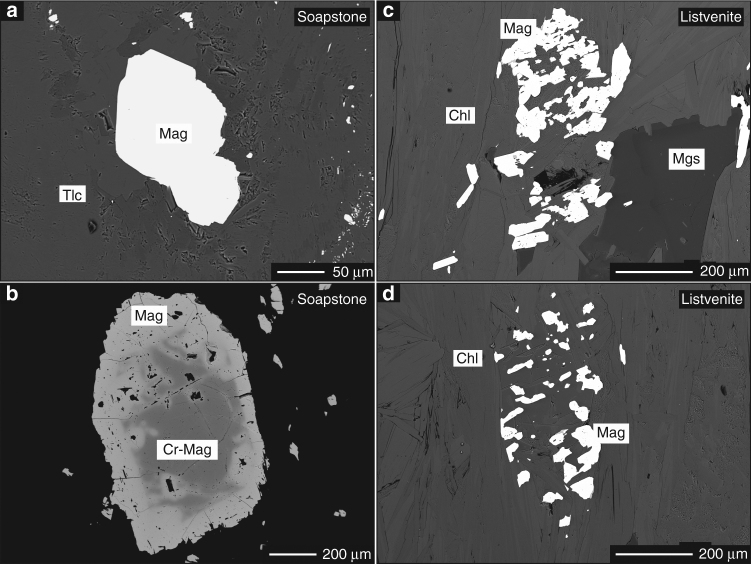
Reaction textures of magnetic carrier minerals. BSE images showing a comparison of magnetite textures between soapstone **a** and **b** and listvenite **c** and **d**. Magnetite in the soapstone is coarse grained and exhibits a subhedral crystal shape. In contrast, magnetite in the listvenite is usually fine grained with individual grains forming clusters that outline the size and shape of magnetite in the soapstone. Soapstone magnetite typically contains a chromium–bearing magnetite (Cr–Mag) core **b**

SQUID microscopy on LUC thin section samples support the outcrop-scale magnetic anomaly behavior in the serpentinite, soapstone, and listvenite assemblages: high (~6.7 µT) NRM magnetic field values in serpentinite, even higher (~9.4 µT) in the soapstone and almost none (except for a few grains of 1.8–3.0 µT per measured sample) in the listvenite (Fig. 7a). Petrographic observations confirm that these samples are consistent with previously described LUC field samples^13, 26^. CO_2_ mass fractions of each sample used for thin section preparation were measured as ~2.35 wt%, ~14.9 wt%, and ~29.3 wt% for serpentinite, soapstone, and listvenite, respectively, (Supplementary Table 1)^13^. As observed in outcrop and thin section, listvenite formation can be heterogeneous in terms of mineral composition and reaction progress, which correlates with the breakdown of magnetite (e.g., Fig. 8). Listvenite samples–16 and –11 exemplify different stages of reaction progress (Fig. 7a). Sample listvenite–11 is completely altered and contains sulfide minerals and is devoid of magnetite, while listvenite–16 contains talc and magnetite relicts and represents incomplete alteration. The ARM magnetic field values of both listvenite samples exhibit this heterogeneity in the weakest magnetic field values among the two carbonation product assemblages. Magnetite inclusions in thermodynamically stable soapstone-magnesite are effectively passivated from further replacement reactions and together with rare sulphide minerals contribute to the weak magnetic field strength of the listvenite samples. Overall, ARM magnetic field values follow the same trend as the NRM, confirming that (i) the observed NRM in the thin section samples reflects a lithology dependent (i.e., abundance of magnetic carriers) magnetic source; (ii) a strong correlation exists with the amplitude and wavelength variations in magnetic field values; and (iii) NRM in our field samples reflects the behavior observed in total-field anomaly profiles and further link the observed changes in magnetic anomaly amplitudes and wavelengths to mineral carbonation reactions at the grain scale (Fig. 7a, b).

### Replacement reactions involving magnetic carrier minerals

**Fig. 9:**
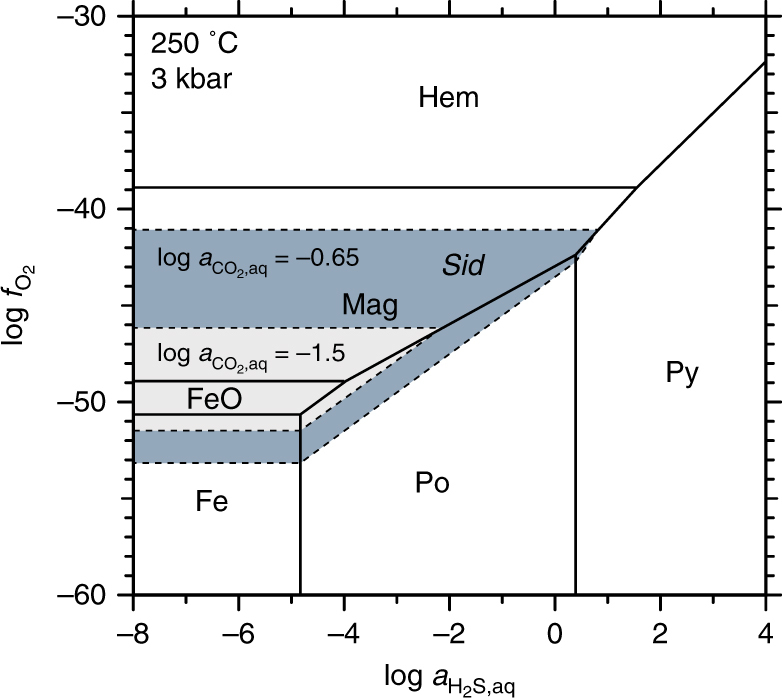
Thermodynamic stability of magnetic signal carrier minerals. H_2_S–O_2_ activity diagram in the system Fe–O_2_–H_2_S showing the stability of native iron, iron oxide and iron sulfide phases as a function of fluid H_2_S activity and oxygen fugacity. The shaded areas indicate stability of siderite over native iron, iron oxide and iron sulfide phases at fluid CO_2_ activities corresponding to soapstone (gray field) and listvenite (dark gray field) formation (hexagon symbols in Fig. 3). The magnetite stability field is significantly reduced relative to siderite at a fluid CO_2_ activity that stabilizes the listvenite assemblage. The diagram was calculated using the computer program Supcrt and thermodynamic database dprons96.dat^50^. The same diagram calculated for alteration temperatures of 180 °C and 300 °C is included in the supplement (Supplementary Fig. 2)

Variations in the magnetic signal strength of the different alteration assemblages can be linked to the stability of magnetic carrier minerals at the relevant alteration conditions. Thermodynamic models and hydrothermal experiments predict an increasing abundance of magnetite during the alteration sequence from peridotite serpentinization, intermittently formed soapstone, to listvenite^10, 23, 24^. As a result, total magnetic field intensity is expected to increase along the isothermal carbonation reaction path, while the absolute magnetic field intensity value depends on the amount of magnetite formed and thus on alteration temperature. In the serpentinite and soapstone samples, magnetite is the only mineral phase contributing to the magnetic signal, whereas listvenite sometimes contains additional sulfide minerals (pyrite, chalcopyrite, pyrrhotite) (Fig. 2f–h). Magnetite precipitates and dissolves depending on the availability of dissolved Fe and its thermodynamic stability along the reaction path (Figs 3, 9)^32^ and the distribution of Fe between secondary phases is controlled by their Fe–Mg exchange potentials (Δµ(Fe^2+^Mg_−1_))^23^. For the dominant silicate phases involved in ultramafic rock carbonation, preferential uptake of Fe is in the order: olivine > antigorite > talc^33, 34^. Alteration of peridotite to form serpentinite and soapstone is therefore accompanied by release of Fe that is not partitioned into secondary serpentine and talc but available for magnetite formation:

3 $$3{\mathrm{FeO}} + 0.5\,{\mathrm{O}}_2 \to {\mathrm{Fe}}_3{\mathrm{O}}_4.$$

The increase in magnetite abundance and the composition of secondary silicate (talc X_Mg_ ≈ 0.95; chlorite X_Mg_ ≈ 0.92) and carbonate phases (magnesite X_Mg_ ≈ 0.93) in soapstone relative to serpentinite (antigorite X_Mg_ ≈ 0.94) is thus consistent with the thermodynamic prediction and reflected by magnetic field intensity variations in outcrop and thin section-scale magnetic mapping across serpentinite-soapstone interfaces (Figs. 6b, 7a).

However, decreasing magnetic field intensity across the soapstone-listvenite front (Fig. 6e) and the breakdown of magnetite in listvenite as observed in thin section analysis (Fig. 7a) and e.g., by Hansen et al.^16^ contradicts model predictions^24, 32^. This suggests that listvenite formation involves formation of non-magnetic minerals at the expense of magnetite. The presence of magnetite in the samples prior to listvenite formation is supported by dissolution textures of oxide phases present as small individual grains of <10 µm in diameter, forming clusters mimicking the size and shape of larger precursor grains identical to those present in the soapstone (Fig. 8). Furthermore, magnetite inclusions in the core of listvenite-magnesite indicate its stability in the soapstone prior to the growth of inclusion–free, Fe–enriched magnesite rims during listvenite formation (Figs. 4f, 5). Magnetite breakdown releases two Fe^3+^ ions for each Fe^2+^ ion and additional small amounts of Fe^3+^ may be released from breakdown of serpentine and talc. Secondary sheet silicate (talc, mica, and chlorite), carbonate (as siderite component), and sulfide (pyrite, pyrrhotite) phases predominantly incorporate Fe^2+^, while talc and chlorite may take up small amounts of Fe^3+^. At the high fluid CO_2_ activities required to stabilize the listvenite assemblage, siderite forms at the expense of magnetite thereby effectively reducing the released ferric Fe (Fig. 9):

4 $${{{{\rm{Fe}}_{\rm 3}{\rm{O}}_{\rm 4}}}\atop{{\rm{Magnetite}}}} + {\rm 3}\,{\rm{CO}}_{\rm{2}} \to {{{\rm 3}\,{\mathrm{FeCO}}_{\rm 3}}\atop{\mathrm{Siderite}}} + 0.5\,{\mathrm{O}}_2.$$

The change in iron oxidation state during serpentinite carbonation is reflected by bulk rock Fe^2+^/Fe^3+^ of ~1.19 in serpentinite, ~0.86 in soapstone, and ~11.5 in the listvenite (Supplementary Table 1)^13^. Siderite represents the Fe component in Fe enriched and magnetite-free listvenite-magnesite overgrowing earlier formed low Fe/Mg soapstone-magnesite. Magnetite breakdown is likely enhanced by reductive dissolution in the presence of dissolved reduced sulfur species (H_2_S and HS^−^)^35, 36^, providing charge balance and sulfur for the formation of sulfide phases in the listvenite (Figs. 2h, 9). Textural observations reveal that sulfide-bearing listvenite is almost devoid of magnetite whereas magnetite relicts are commonly preserved in samples without sulphide (Figs. 4f, 8c, d). Involvement of reduced organic carbon in driving ferric iron reduction is ruled out based on the ^13^C enriched isotopic signature of magnesite^13, 37, 38^.

## Discussion

The baseline for magnetic signal changes during serpentinite carbonation is defined by the magnetite content of the serpentinite and is strongly dependent on the serpentinization progress and temperature, in addition to the composition of the precursor peridotite (e.g., variation in orthopyroxene content) and alteration fluid (e.g., silica activity)^39–43^. Hence, the magnetic signal of different serpentinite occurrences is likely to be different from the LUC^43^. The stability of magnetite during subsequent carbonation is also dependent on the alteration fluid composition and temperature, which has been higher at the LUC than the inferred optimal carbonation temperature of olivine and heat-treated serpentine minerals^3, 12^. At a lower carbonation temperature silicate minerals may incorporate more iron thus reducing the amount of magnetite formed. However, carbonation-related changes in magnetite abundance at different temperatures will still allow for monitoring of the carbonation reaction progress even if absolute magnetic field strength and susceptibility of the starting material are offset to higher or lower values relative to this study (Supplementary Figs. 1, 2).

The aeromagnetic and field measurements, supported by SQUID microscopy and petrographic and geochemical assessment of the rock samples, allow us to draw a strong correlation between reaction-induced changes in the abundance of magnetic minerals and the amplitude, wavelength and wavelength variations in the remotely observed magnetic field values. The strong correlation of changes in magnetic field intensity with increasing bulk rock CO_2_ mass fractions from serpentinite (~2.35 wt%) to soapstone (~14.9 wt%) and to listvenite (~29.3 wt%) demonstrates that measureable geophysical signals are associated with changes in mineralogy during the carbonation sequence (Fig. 7b). The breakdown of magnetite during listvenite formation is consistent with observations at other locations^16, 44, 45^ suggesting that the decrease in magnetic field intensity is a common consequence of intense ultramafic rock carbonation. These observations imply that remote magnetic sensing can be effectively utilized for delineating the extent and degree of serpentinite carbonation and investigating active carbonation reactions through time by periodically measuring the static magnetic field intensity, e.g., at magnetic stations and in boreholes. While natural serpentinite carbonation may take place during metamorphic cooling, the isothermal reaction path of the LUC is likely consistent with CO_2_ mitigation schemes as cooling rates in relatively deep situated ultramafic target rocks are slow relative to the required CO_2_ injection rates and the exothermic carbonation reaction may balance cooling resulting from fluid injection^3^. Furthermore, knowledge of carbonation temperature and fluid pressure at depth can be obtained from injection well measurements and provide crucial parameters for the correct interpretation of magnetic signal changes.

## Methods

### Electron probe micro analysis

Quantitative elemental maps were acquired on a JEOL 8530 F electron microprobe equipped with 5 tunable wavelength dispersive spectrometers. Operating conditions were 40° takeoff angle, and a beam energy of 15 keV. The beam current was 20 nA for calibration and map acquisition. The beam diameter was 2 µm. Dwell time was 40 ms per pixel with a pixel dimension of 2 × 2 µm. Elements were acquired using analyzing crystals LiFH for Ti Kα1, Cr Kα1, Mn Kα1, LiF for Fe Kα1, Ni Kα1, PETJ for Ca Kα1, K Kα1, and TAP for Mg Kα1, Si Kα1, Al Kα1, and Na Kα1. The standards were an assortment of synthetic and natural minerals and metals. The counting time was 20 s on peak for all elements, and Mean Atomic Number background corrects were used throughout^46^. The intensity data were corrected for Time Dependent Intensity (TDI) loss (or gain) using a self-calibrated correction for Si Kα1, Na Kα1, Ti Kα1, K Kα1, Fe Kα1. Interference corrections were applied to Fe for interference by Mn, and to Mn for interference by Cr^47^. Results are the average of three points and detection limits ranged from 0.006 wt% for Si Kα1 to 0.008 wt% for Al Kα1 to 0.009 wt% for Na Kα1 to 0.012 wt% for Ti Kα1 to 0.028 wt% for Ni Kα1. Oxygen was calculated by cation stoichiometry and included in the matrix correction. The elemental maps were processed using Probe Software’s CalcImage application. The matrix correction method was ZAF and the mass absorption coefficients data set was LINEMU Henke (LBL, 1985) < 10KeV/CITZMU >10KeV. The ZAF algorithm utilized was Armstrong/Love Scott^48^.

### Whole-rock geochemical analyses

Whole-rock geochemical analyses including CO_2_ and FeO were performed by Actlabs Laboratories Ltd., using the lithium metaborate/tetraborate fusion ICP Whole Rock and the trace element ICP/MS packages.

Samples are mixed with a flux of lithium metaborate and lithium tetraborate and fused in an induction furnace. The melt is immediately poured into a solution of 5% nitric acid containing an internal standard, and mixed continuously until completely dissolved (~30 min). The samples are run for major oxides and selected trace elements on a combination simultaneous/sequential Thermo Jarrell–Ash ENVIRO II ICP or a Varian Vista 735 ICP. Calibration is performed using 7 prepared USGS and CANMET certified reference materials. One of the 7 standards is used during the analysis for every group of ten samples. FeO is determined through titration, using a cold acid digestion of ammonium metavanadate, and hydrofluoric acid in an open system. Ferrous ammonium sulphate is added after digestion and potassium dichromate is the titrating agent. Weight fractions of dry CO_2_ sample gas are measured by infrared absorption after decomposing 0.2 g of sample material in a resistance furnace in a pure nitrogen environment at 1000 °C, using an ELTRA CW–800 (www.actlabs.com).

### Data availability

All the data generated or analyzed during this study are included in this published article (and its Supplementary Information files). Samples and data used in this study are available through MAPLES (Multiscale Applied Physics Lab for Earth Science) at Department of Geology and Geophysics, Texas A&M University via email contact (masako.tominaga@tamu.edu).

## Electronic supplementary material

Supplementary Information
