# Supplementary material for: Multi-scale magnetic mapping of serpentinite carbonation
Source: Nat Commun. 2017 Nov 30;8:1870. doi: 10.1038/s41467-017-01610-4 (PMC5709373; doi:10.1038/s41467-017-01610-4)
Supplement: Supplementary file 1 — Supplementary Information [file 41467_2017_1610_MOESM1_ESM.pdf]

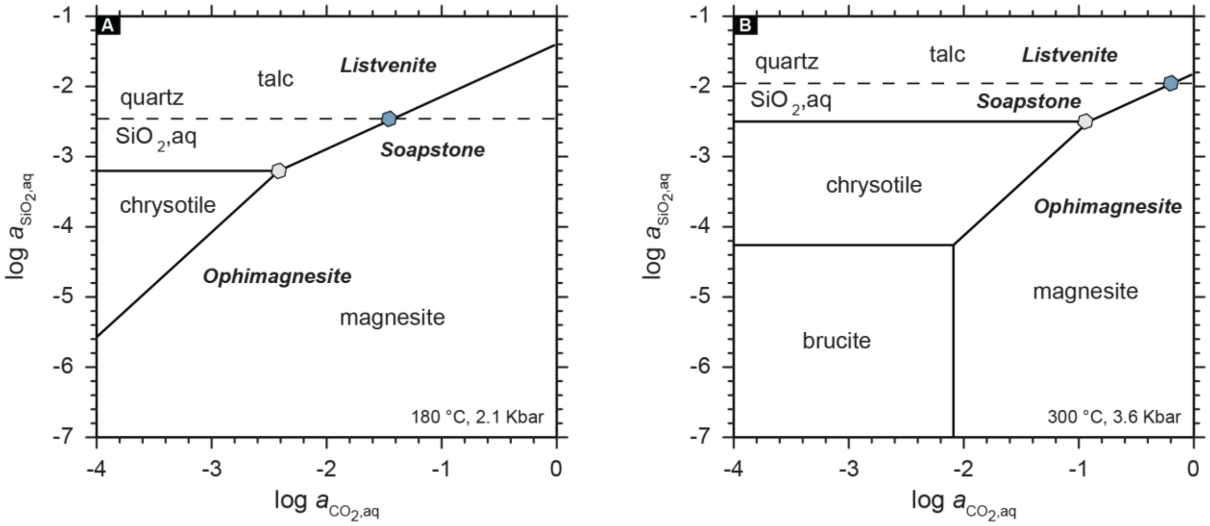

Supplementary Figure 1 Reaction path of serpentinite carbonation. CO_2–SiO_2 activity diagrams in the system MgO–SiO_2–H_2O–CO_2 showing the isothermal and isobaric reaction path of progressive serpentinite carbonation resulting in soapstone (talc + magnesite) and listvenite (quartz + magnesite) formation at 180 °C and 2.1 kbar (A) and 300 °C and 3.6 kbar (B). An optimum peridotite carbonation temperature of 180 °C has been suggested by Kelemen and Matter [1]. Pressure values were chosen based on a thermobarometric gradient of 0.0125 kbar/°C. Hexagon symbols mark CO_2 activity values used for the calculation of Supplementary Figure 2. Mineral stability fields are calculated using the computer program Supcrt and thermodynamic database dprons96.dat [2], quartz saturation is based on the thermodynamic data of Rimstidt [3].

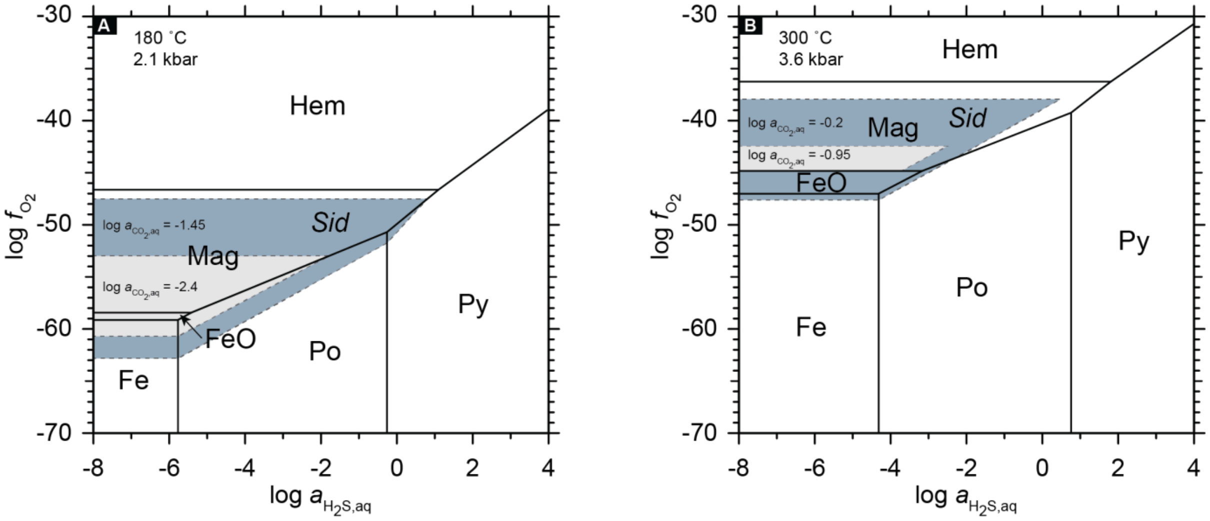

Supplementary Figure 2 Thermodynamic stability of magnetic signal carrier minerals. H_2S–O_2 activity diagrams in the system Fe–O_2–H_2S showing the stability of iron, iron oxide and iron sulfide phases as a function of fluid H_2S activity and oxygen fugacity at 180 °C and 2.1 kbar (A) and 300 °C and 3.6 kbar (B). The shaded fields show the stability of siderite at fluid CO_2 activities corresponding to soapstone and listvenite formation (hexagon symbols in Supplementary Figure 1). The magnetite stability field is significantly reduced relative to siderite at a fluid CO_2 activity that stabilizes the listvenite assemblage. The diagram was calculated using the computer program Supcrt and thermodynamic database dprons96.dat [2].

Supplementary Table 1 Bulk rock geochemical data and calculated magnetite mass fraction of serpentinite, ophimagnesite, soapstone and listvenite samples from the LUC.

	**serpentinte** n = 9		**ophimagnesite** n = 9		**soapstone** n = 18		**average listvenite** n = 5	
	wt%	1σ	wt%	1σ	wt%	1σ	wt%	1σ
**SiO_2**	40.08	2.02	39.62	3.56	35.15	2.48	25.09	9.71
**Al_2O_3**	2.43	0.98	2.70	1.06	1.58	0.80	3.42	0.40
**Cr_2O_3**	0.82	0.30	0.94	0.25	0.82	0.24	0.78	0.12
**Fe_2O_3 total**	8.25	n.c.	7.96	n.c.	8.45	n.c.	9.26	n.c.
**Fe_2O_3**	3.56	1.44	3.56	0.91	4.32	0.93	0.67	0.32
**FeO**	4.22	0.54	3.96	0.60	3.72	0.43	7.73	1.07
**MnO**	0.09	0.01	0.09	0.04	0.12	0.03	0.13	0.02
**MgO**	34.97	1.33	33.05	2.47	32.73	1.34	27.62	4.65
**CaO**	1.01	0.58	0.97	0.49	1.85	1.71	1.83	0.97
**Na_2O**	0.02	0.00	0.02	0.01	0.02	0.01	0.38	0.28
**K_2O**	0.01	0.00	b.d.l.	n.c.	0.01	b.d.l.	n.c.	0.22
**TiO_2**	0.07	0.07	0.10	0.07	0.06	0.07	0.23	0.03
**P_2O_5**	0.02	0.01	0.02	0.00	0.02	0.01	0.02	0.00
**NiO**	0.23	0.04	0.23	0.03	0.23	0.06	0.17	0.04
**CO_2**	2.35	0.79	6.87	1.74	14.88	2.21	29.34	6.05
**H_2O**	9.68	0.71	7.05	1.17	3.94	0.93	0.64	0.66
**Total**	99.50	0.71	99.15	0.66	99.44	0.74	98.23	0.16
**Fe^{2+}/Fe^{3+}**	1.19		1.11		0.86		11.50	
**wt% Mag**	5.16		5.16		6.27		0.97	

*b.d.l. = below the detection limit; n.c. = not calculated

References

1. Kelemen PB, Matter J. In situ carbonation of peridotite for CO_2 storage. *P Natl Acad Sci USA* 105, 17295-17300 (2008).
2. Johnson JW, Oelkers EH, Helgeson HC. SUPCRT92: A software package for calculating the standard molal thermodynamic properties of minerals, gases, aqueous species, and reactions from 1 to 5000 bar and 0 to 1000 °C. *Comput Geosci* 18, 899-947 (1992).
3. Rimstidt JD. Quartz solubility at low temperatures. *Geochim Cosmochim Ac* 61, 2553-2558 (1997).
